# Malignant peripheral nerve sheath tumor arising from the greater omentum: Case report

**DOI:** 10.1186/1477-7819-9-33

**Published:** 2011-03-21

**Authors:** Masashi Miguchi, Yuji Takakura, Hiroyuki Egi, Takao Hinoi, Tomohiro Adachi, Yasuo Kawaguchi, Manabu Shinomura, Masakazu Tokunaga, Masazumi Okajima, Hideki Ohdan

**Affiliations:** 1 Deparment of Gastroenterological Surgery, Hiroshima University Hospital 1-2-3 Kasumi, Minami-ku, Hiroshima city, Hiroshima 734-8551, Japan

## Abstract

Malignant peripheral nerve sheath tumors (MPNSTs) are rare soft tissue tumors that arise from a peripheral nerve or exhibit nerve sheath differentiation. Most of these tumors arise on the trunk, extremities, or head and neck regions; they are very rarely located in the abdominal cavity. The patient was a 71-year-old man who was referred to our hospital for a mass and pain in the right lower abdomen. Abdominal computed tomography revealed a large (9 × 9 cm), well-circumscribed, lobulated, heterogeneously enhanced mass in the pelvis. Exploratory laparotomy revealed a large mass in the greater omentum, and the tumor was completely excised. Histopathological analysis revealed that the tumor was composed of spindle cells with high mitotic activity. On staining the tumor, positive results were obtained for S-100 but negative results were obtained for c-kit, cluster of differentiation (CD)34, α-smooth muscle actin, and desmin. These findings strongly supported a diagnosis of MPNST primarily arising from the greater omentum. To the best of our knowledge, this is the first reported case of an MPNST arising from the greater omentum. In this report, we have described the case of a patient with an MPNST arising from the greater omentum and have discussed the clinical characteristics and management of MPNSTs.

## Background

Primary solid omental tumors are rare and include various types of tumors such as gastrointestinal stromal tumors (GIST), leiomyosarcomas, hemangiocytomas, fibrosarcomas, leiomyomas, liposarcomas, desmoids tumors, fibromas, mesotheliomas, and myosarcomas [1]. Although the pathological spectrum of primary omental tumors is diverse, no report has yet been published on malignant peripheral nerve sheath tumors (MPNSTs) arising from the greater omentum.

In this report, we describe the extremely rare case of a Japanese man who had an MPNST arising from the greater omentum.

## Case presentation

**Figure 1:**
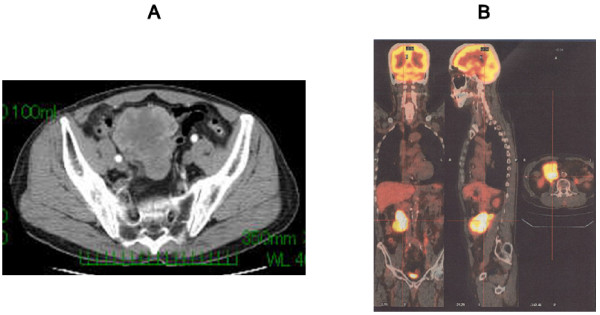
**Preoperative imaging findings**. **A**) CT scan shows a large, well-circumscribed, lobulated mass in the pelvis. The central region of the mass appears to have low density, while the marginal region is well enhanced. **B**) FDG-PET/CT shows a mass with increased FDG accumulation in the right lower abdomen, without any evidence of distant metastasis.

The patient was a 71-year-old man who was healthy by birth and was admitted to our hospital with pain in the right lower abdomen. Physical examination revealed a large, firm, movable mass in the abdomen. The hematological tests, including those for the serum levels of tumor markers such as carcinoembryonic antigen (CEA), carbohydrate antigen (CA) 19-9, and CA125, yielded normal results. Abdominal computed tomography (CT) revealed a large (approximately, 9 × 9 cm), well-circumscribed, lobulated mass in the pelvis. The central region of the mass appeared to have low density, while the marginal region was well enhanced in the CT scan (Figure 1A). CT/positron emission tomography (PET) with ^18^F-fluorodeoxyglucose (FDG) showed a mass with increased FDG accumulation in the right lower abdomen, without any evidence of distant metastasis (Figure 1B). Evaluation of the gastrointestinal tract did not yield any definite results. The origin of the tumor could not be clearly determined.

**Figure 2:**
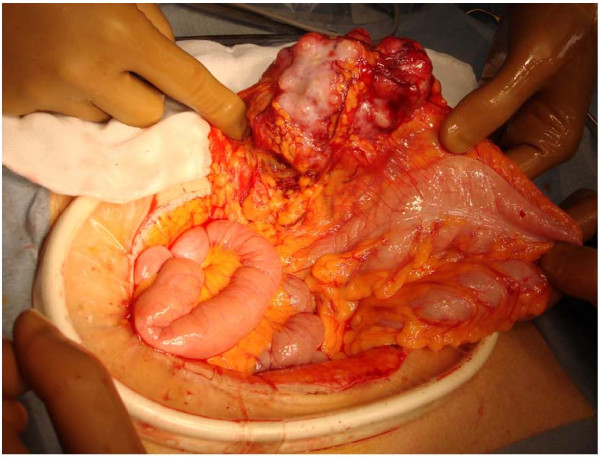
**The tumor arises from the greater omentum and is not connected with the gastrointestinal tract**.

Exploratory laparotomy was performed under the diagnosis of an intra-abdominal tumor of unknown origin. During laparotomy, it was observed that the tumor arose from the greater omentum and was not connected with the gastrointestinal tract (Figure 2). The tumor was completely excised along with the greater omentum.

**Figure 3:**
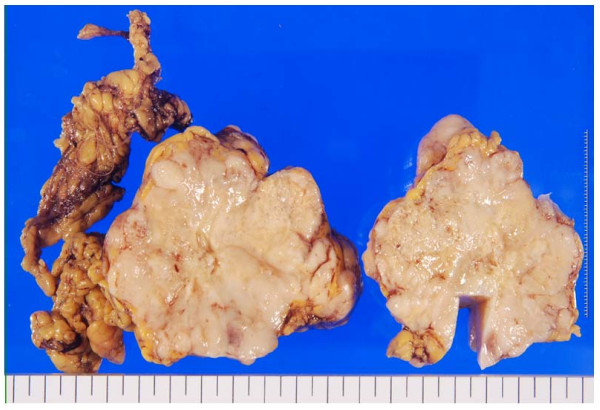
**Macroscopically, the tumor is whitish grey and is relatively firm and solid**.

**Figure 4:**
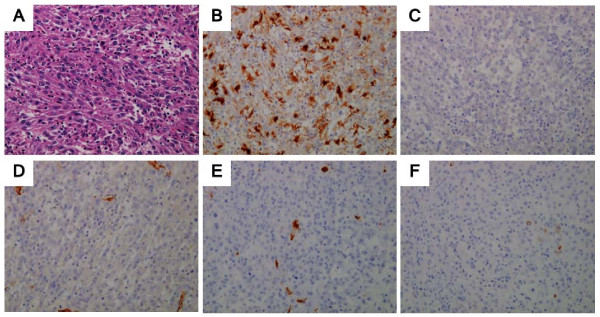
**Microscopic analysis (A: hematoxylin-eosin (HE) stain; B, C, D, E, F: immunohistochemical analysis)**. **A**: Spindle cells arranged in intersecting fascicles and polygonal cells arranged in sheets grow infiltrating. The cellular nuclei are polygonal (bulky, roundish, and irregular), and the tumor cells show 150 mitoses per 50 high-power fields (HE stain; magnification, ×20). **B, C, D, E, F**: Immunohistochemical images show positive staining of tumor cells for S-100 but negative staining for c-kit, α-SMA, desmin, and CD34. (B: S-100, C: c-kit, D: α-SMA, E: desmin, F: CD34)

Gross pathological examination revealed that the tumor was a whitish-grey oval mass, with a maximum diameter of 9 cm (Figure 3). Microscopic examination revealed spindle cells arranged in intersecting fascicles and polygonal cells arranged in sheets grow infiltrating (Figure 4A). The cellular nuclei were polygonal (bulky, roundish, and irregular), and the mitotic activity was 150 mitoses per 50 high-power fields. Coagulative necrosis and myxoid changes were observed in the tumor. Immunohistochemical analysis of the tumor cells yielded positive staining results for S-100 (Figure 4B) but negative results for c-kit, α-smooth muscle actin (α-SMA), desmin, and cluster of differentiation (CD)34 (Figure 4C-F). The morphology and immunoprofile of the tumor strongly supported a diagnosis of MPNST.

After an uneventful postoperative course, the patient was discharged on the ninth postoperative day. At 12 months after surgery, the patient was in good condition, and no evidence of local recurrence or distant metastases was noted.

## Discussion

MPNSTs are rare soft tissue tumors that arise in proximity to large peripheral nerves and account for 3-10% of all soft tissue sarcomas [2,3]. The term MPNST was coined by the World Health organization (WHO) and is defined as any tumor that arises from a peripheral nerve; this term replaces previously used heterogeneous and often confusing terminology, such as malignant schwannoma, malignant neurilemmoma, and neurofibrosarcoma, for tumors of neurogenic origin and similar biological behavior. These tumors arise from major or minor peripheral nerve branches or from the sheath of peripheral nerve fibers. Most of these tumors arise on the trunk, extremities, or the head and neck region [4,5]. MPNSTs arising from the abdominal cavity are extremely rare. Only a few cases of MPNSTs arising from the gastrointestinal tract have been reported [6,7], and to date, no cases of MPNSTs arising from the greater omentum have been reported in the literature. Although 4 cases of "benign schwannoma" of the greater/lesser omentum have been reported in earlier studies [8-11], high mitotic activity, which indicates malignant potential, was noted only in our patient. Therefore, to the best of our knowledge, this is the first reported case of an MPNST arising from the greater omentum.

The pathologic diagnosis of MPNST is facilitated by features such as palisading arrangement, nuclear atypia, bizarre giant cells, mitotic figures, and necrosis. These tumors have morphological heterogeneity, and staining analysis of such tumors reveals spindle cells with a fascicular pattern [12]. Histological and immunohistochemical markers specific for MPNSTs are not available. The S100 protein is the antigen most commonly used to identify nerve sheath tumors of various types. However, S100 protein immunoreactivity is detected in only 50-60% of MPNSTs, and this protein is also expressed in a range of other tissues and tumor types [13,14]. Different markers are used to exclude other spindle cell tumors. Desmin and α-SMA are used to exclude smooth muscle tumors, and CD34 and CD117 (c-kit) are used to exclude GIST [15]. In our case, the strong S-100 expression without expression of other immunohistochemical markers indicated the presence of an MPNST.

To date, little is known about MPNSTs arising from the abdominal cavity. Therefore, the prognosis of and initial treatments for such tumors are uncertain. A recently published study investigated the overall prognostic factors and survival of patients with MPNSTs in all locations [4,5]. The results of this study, which involved patients with localized MPNSTs, suggested that the disease-specific survival rate for MPNSTs was around 50% at 5 years. Most clinical series reported that tumor size was the most reliable independent prognostic factor; larger tumor size was related with worse outcome. Zou et al. reported that negative staining results for S-100 were associated with prognosis when the tumors were completely resected [5].

Survival appears to be related to complete tumor resection. Therefore, complete surgical resection of the tumor in patients with MPNSTs is of utmost importance for their treatment.

It remains uncertain whether chemotherapy and radiotherapy have a positive impact on the survival of patients with MPNSTs. The results of most case series indicate limited benefits and high morbidity on using adjuvant radiotherapy or chemotherapy. Despite aggressive combined radiation and systemic chemotherapy, the 5-year survival rates for MPNSTs range from 35% to 50% [16,17]. The current recommendation is that this therapy be reserved for recurrent tumors, suspected residual microscopic disease, and high-grade tumors [7].

Although these data may only describe what is known regarding the behavior of this tumor in other locations of the body, we recommend wide excision of MPNSTs with very close postoperative follow-up imaging.

## Conclusion

MPNSTs arising from the greater omentum are extremely rare. It is important to recognize that an abdominal mass may be caused by an MPNST. MPNSTs should be considered as a rare differential diagnosis for a tumor in the greater omentum.

Because no definite microscopic criteria are available for distinguishing between benign and malignant tumors, radical excision is the treatment of choice for MPNSTs, and prolonged follow-up is essential.

## Consent

Written informed consent was obtained from the patient for publication of this case report and any accompanying images. A copy of the written consent is available for review by the Editor-in-Chief of this journal

## Abbreviations

CA: carbohydrate antigen; CEA: carcinoembryonic antigen; CT: computed tomography; FDG: fluorodeoxyglucose; GIST: gastrointestinal stromal tumor; MPNST: malignant peripheral nerve sheath tumor; PET: positron emission tomography; WHO: World Health Organization; α-SMA: α-smooth muscle actin.

## Competing interests

The authors declare that they have no competing interests.

## Authors' contributions

MM participated in treatment of the patient, collected case details, literature search and draft the manuscript. YT participated in treatment of the patient and helped to draft the manuscript. HE, TH, TA, YK, MS, MT and MO participated in treatment of the patients. HO participated in treatment planning of the patient and helped to draft the manuscript. All authors read and approved the final manuscript.
